# Impact of Preoperative Frailty on Outcomes in Patients with Cervical Spondylotic Myelopathy Undergoing Anterior vs. Posterior Cervical Surgery

**DOI:** 10.3390/jcm13010114

**Published:** 2023-12-25

**Authors:** Aladine A. Elsamadicy, Sumaiya Sayeed, Josiah J. Z. Sherman, Samuel Craft, Benjamin C. Reeves, Sheng-Fu Larry Lo, John H. Shin, Daniel M. Sciubba

**Affiliations:** 1 Department of Neurosurgery, Yale University School of Medicine, New Haven, CT 06510, USA; 2 Department of Neurosurgery, Zucker School of Medicine at Hofstra, Long Island Jewish Medical Center, North Shore University Hospital, Northwell Health, Manhasset, NY 11030, USA; 3 Department of Neurosurgery, Massachusetts General Hospital, Harvard Medical School, Boston, MA 02114, USA

**Keywords:** frailty, cervical spondylotic myelopathy, ACDF, PCDF, length of stay

## Abstract

**Introduction**: Frailty has been shown to negatively influence patient outcomes across many disease processes, including in the cervical spondylotic myelopathy (CSM) population. The aim of this study was to assess the impact that frailty has on patients with CSM who undergo anterior cervical discectomy and fusion (ACDF) or posterior cervical decompression and fusion (PCDF). **Materials and Methods**: A retrospective cohort study was performed using the 2016–2019 national inpatient sample. Adult patients (≥18 years old) undergoing ACDF only or PCDF only for CSM were identified using ICD codes. The patients were categorized based on receipt of ACDF or PCDF and pre-operative frailty status using the 11-item modified frailty index (mFI-11): pre-Frail (mFI = 1), frail (mFI = 2), or severely frail (mFI ≥ 3). Patient demographics, comorbidities, operative characteristics, perioperative adverse events (AEs), and healthcare resource utilization were assessed. Multivariate logistic regression analyses were used to identify independent predictors of extended length of stay (LOS) and non-routine discharge (NRD). **Results**: A total of 37,990 patients were identified, of which 16,665 (43.9%) were in the pre-frail cohort, 12,985 (34.2%) were in the frail cohort, and 8340 (22.0%) were in the severely frail cohort. The prevalence of many comorbidities varied significantly between frailty cohorts. Across all three frailty cohorts, the incidence of AEs was greater in patients who underwent PCDF, with dysphagia being significantly more common in patients who underwent ACDF. Additionally, the rate of adverse events significantly increased between ACDF and PCDF with respect to increasing frailty (*p* < 0.001). Regarding healthcare resource utilization, LOS and rate of NRD were significantly greater in patients who underwent PCDF in all three frailty cohorts, with these metrics increasing with frailty in both ACDF and PCDF cohorts (LOS: *p* < 0.001); NRD: *p* < 0.001). On a multivariate analysis of patients who underwent ACDF, frailty and severe frailty were found to be independent predictors of extended LOS [(frail) OR: 1.39, *p* < 0.001; (severely frail) OR: 2.25, *p* < 0.001] and NRD [(frail) OR: 1.49, *p* < 0.001; (severely frail) OR: 2.22, *p* < 0.001]. Similarly, in patients who underwent PCDF, frailty and severe frailty were found to be independent predictors of extended LOS [(frail) OR: 1.58, *p* < 0.001; (severely frail) OR: 2.45, *p* < 0.001] and NRD [(frail) OR: 1.55, *p* < 0.001; (severely frail) OR: 1.63, *p* < 0.001]. **Conclusions**: Our study suggests that preoperative frailty may impact outcomes after surgical treatment for CSM, with more frail patients having greater health care utilization and a higher rate of adverse events. The patients undergoing PCDF ensued increased health care utilization, compared to ACDF, whereas severely frail patients undergoing PCDF tended to have the longest length of stay and highest rate of non-routine discharge. Additional prospective studies are necessary to directly compare ACDF and PCDF in frail patients with CSM.

## 1. Introduction

Degenerative cervical spondylosis is a common cause of disability in the elderly, with existing studies suggesting a prevalence of radiographic disc herniation in up to 90% of people over 50 years old [1]. Herniated discs, osteophytes, ossified ligaments, and other degenerative changes implicated in cervical spondylosis may cause neck pain and impinge on the spinal cord, producing neurologic dysfunction [2,3,4]. Cervical spondylotic myelopathy (CSM) frequently requires surgical intervention, with both anterior cervical discectomy and interbody fusion (ACDF) and posterior cervical decompression and fusion (PCDF) being commonly performed approaches, though debate remains as to which approach may be superior [5,6]. Given the recent increase in surgical intervention for CSM and the expected aging of the United States’ population [7,8,9], additional studies are necessary to better understand how patient risk-factors impact outcomes within these different surgical approaches.

Frailty, characterized by a reduced physiological reserve, encompasses a wide range of systemic and physiological health outcomes related to the loss of skeletal muscle mass (sarcopenia), reduced bone quality, cognitive dysfunction, and immune system impairment [10,11,12]. Given the physiological complexity and clinical relevance, frailty decision-making tools have been developed to efficiently identify frailty and predict patient outcomes; the modified frailty index (mFI) is such a tool developed using the American College of Surgeons National Surgical Quality Improvement Program (ACS-NSQIP) that has gained widespread use across medical specialties [13,14]. In spine surgery, both the mFI-5 and mFI-11 have been assessed for their ability to identify frailty and predict poor outcomes in patients undergoing surgery for degenerative spine disease [15,16], adult spinal deformity [17], spinal metastases [18], and other disorders [19]. Given the risk of perioperative complications and high healthcare resource utilization in this population [6,20,21,22], studies assessing how frailty impacts patients with degenerative CSM undergoing ACDF or PCDF is necessary for better risk assessment and surgical decision making [23].

The aim of this study was to investigate the impact of preoperative frailty on patients with CSM who undergo ACDF vs. PCDF.

## 2. Materials and Methods

### 2.1. Data Source and Patient Population

The national inpatient sample (NIS) database is a stratified discharge database from the Healthcare Cost and Utilization Project (HCUP). The NIS represents 20% of all inpatient admissions from community hospitals in the United States. It is the largest all-payer healthcare database in the US, containing over 7 million hospital admissions (approximately 35 million hospitalizations, weighted) per year. A retrospective study was performed using years 2016, 2017, 2018, and 2019 of the NIS for all adult (≥18 years old) ACDF or PCDF for CSM. The Institutional Review Board was deemed exempted due to the deidentification of patients in the NIS database. As all inpatient admissions were deidentified by HCUP, informed consent was deemed exempt as well.

The International Classification of Diseases, Tenth Revision, Clinical Modification (ICD-10-CM) diagnosis and procedural coding system (PCS) was used to identify patients and their respective comorbidities and surgical interventions. Adult patients with a primary diagnosis code of CSM (ICD-10-CM M47.12) were identified. ICD-10-CM procedural codes were then cross-matched to identify patients in the cohort undergoing ACDF (0RG10A0, 0RG20A0) or PCDF (00NW0ZZ, 0RH104Z) (Appendix A Table A1). The patients who underwent procedures with a posterior approach to the anterior column, as well as those who underwent percutaneous or endoscopic procedures, were excluded, along with patients with a history of traumatic spine fracture or spinal neoplasm (Appendix A Table A1). Additionally, patients undergoing both anterior and posterior approaches in the same indexed procedure or hospitalization were excluded.

### 2.2. Modified Frailty Index (mFI)

The mFI is a frailty scoring system that was developed utilizing the NSQIP [24], and a validated 11-point scoring system was created that adds 1 point each for impaired functional status, hypertension, history of chronic obstructive pulmonary disease (COPD) or pneumonia, impaired sensorium, diabetes, myocardial infarction, congestive heart failure (CHF), stroke, transient ischemic attack (TIA), percutaneous coronary intervention (PCI), and peripheral vascular disease (PVD) [13]. While originally developed for NSQIP, Subramanian et al. identified ICD-10 codes for the mFI-11 scoring system that we used for our study (Appendix A) [25]. The patients in our cohort were then identified as pre-frail (mFI = 1), frail (mFI = 2), and severely frail (mFI = 3 or more) [26].

### 2.3. Data Collection

Patient demographics such as age, sex, race, median household income, and insurance provider were all collected from the NIS database. Hospital characteristics such as size by bed volume, region (Northeast, South, Midwest, and West), and type (rural, urban teaching, and urban non-teaching) were also collected. The comorbidities assessed included the 11 comorbidities constituting the mFI and Elixhauser comorbidities, such as deficiency anemias, alcohol use, and paralysis. Other patient characteristics assessed were affective disorder, smoking history, cervicalgia, headache, and dorsalgia (Appendix C Table A3). The data on intraoperative variables such as the number of levels fused and the incidence of cerebrospinal fluid leak or dural tear were also collected (Appendix C Table A3).

The data regarding post-operative complications for each patient were collected by indexing additional diagnoses from the NIS database (Appendix C Table A3). The complications included in the analysis included acute kidney injury, acute post-hemorrhagic anemia, post-operative pain, acute respiratory failure, circulatory complications, mechanical ventilation, nervous system complications, urinary tract infection (UTI), sepsis, dysphagia, mechanical device complication, and displacement of internal fixation device of vertebrae (Appendix C Table A3). In addition, postoperative outcome measures such as hospital length of stay (LOS) and discharge disposition were also assessed. Discharge disposition was classified as routine (patient went home, home with healthcare services), non-routine (patient sent to short-term hospital, skilled nursing facility, intermediate care facility), and other (leaving against medical advice, died in hospital, unknown destination).

### 2.4. Statistical Analysis

The national estimates were calculated using discharge-level weights provided by HCUP. The parametric data were expressed as mean ± standard deviation (SD) and compared via one-way Student’s *t*-test. The nonparametric data were expressed as median (interquartile range) and compared via the Mann–Whitney *U* test. The nominal data were compared with the χ^2^ test. For our primary hypothesis, weighted univariate and multivariate logistic regressions were fitted with extended postoperative hospital LOS (as defined by LOS greater than the 75th percentile for the entire cohort) and non-routine discharge (NRD) disposition as the dependent variable. The patients with “other” discharge were excluded from this portion of the analysis to dichotomize routine vs. NRD. A backward stepwise multivariate logistic regression analysis was used to select variables in the final model, using 0.1 as entry and stay criteria. We forced mFI into the model in view of our primary aim. Age and female sex were also forced into the model due to the biological plausibility for confounding. A *p*-value of less than 0.05 was determined to be statistically significant. The statistical analysis was performed using R Studio, Version 2022.02.4+500 “Prairie Trillium” Release, RStudio Inc., Boston, MA, USA.

## 3. Results

### 3.1. Patient Demographics and Hospital Characteristics

A total of 37,990 patients were identified, of which 16,665 (43.9%) were in the pre-frail cohort, 12,985 (34.2%) were in the frail cohort, and 8340 (22.0%) were in the severely frail cohort, Table 1. Of the pre-frail cohort, 11,655 (70.0%) underwent ACDF and 5010 (30.0%) underwent PCDF, Table 1. Of the frail cohort, 8470 (65.2%) underwent ACDF and 4515 (34.8%) underwent PCDF, Table 1. The patients who underwent PCDF were significantly older than the patients who underwent ACDF across frailty cohorts (*p* < 0.001), and the mean patient age increased with frailty status (*p* < 0.001), Table 1. Race varied significantly with frailty status, with severely frail cohorts containing greater proportions of non-white patients compared to pre-frail and frail cohorts (*p* < 0.001), Table 1. A greater proportion of frail and severely frail patients were in the bottom income quartile compared to pre-frail patients (*p* < 0.001), Table 1. A significantly greater proportion of frail (*p* < 0.001) and severely frail (*p* < 0.019) patients held government insurance, Table 1.

### 3.2. Admission and Patient Comorbidities

The comorbidity burden varied notably between cohorts. In comparing frailty cohorts, the prevalence of a number of comorbidities increased with frailty, Table 2. Within the pre-frail cohort, diabetes (ACDF: 11.2% vs. PCDF: 13.9%, *p* = 0.032), paralysis (ACDF: 1.8% vs. PCDF: 6.8%, *p* < 0.001), and cervicalgia (ACDF: 0.9% vs. PCDF: 1.8%, *p* = 0.042) were significantly more prevalent in patients who underwent PCDF compared to patients who underwent ACDF, Table 2. Conversely, impaired sensorium (ACDF: 18.1% vs. PCDF: 14.3%, *p* = 0.007) and smoking history (ACDF: 12.3% vs. PCDF: 9.3%, *p* = 0.015) were significantly more prevalent in patients who underwent ACDF, Table 2. The prevalence of other comorbidities was similar between pre-frail patients who underwent ACDF or PCDF, Table 2. The comorbidities that were significantly more prevalent among patients who underwent ACDF included hypertension (ACDF: 86.9% vs. PCDF: 83.3%, *p* = 0.039), diabetes (ACDF: 63.2% vs. PCDF: 58.4%, *p* = 0.046), and headache (ACDF: 3.4% vs. PCDF: 1.4%, *p* = 0.013), Table 2. The prevalence of other comorbidities was similar between severely frail patients who underwent ACDF or PCDF, Table 2.

### 3.3. Adverse Events

In comparing frailty cohorts, the incidence of some AEs increased with frailty, including acute kidney injury (*p* < 0.001), acute post-hemorrhagic anemia (*p* = 0.005), post-operative pain (*p* = 0.005), acute respiratory failure (*p* < 0.001), circulatory complications (*p* < 0.001), mechanical ventilation (*p* < 0.001), urinary tract infection (*p* < 0.001), sepsis (*p* < 0.001), and dysphagia (*p* < 0.001), Table 3. Similarly, the incidence of any complication (*p* < 0.001) and the number of complications increased with frailty (*p* < 0.001), Table 3. Within the pre-frail cohort, incidence of any complication (ACDF: 15.4% vs. PCDF: 19.3%, *p* = 0.008) and the number of complications (*p* = 0.021) was greater in patients who underwent PCDF. Dysphagia affected a greater proportion of patients who underwent ACDF (ACDF: 8.3% vs. PCDF: 2.3%, *p* < 0.001), Table 3. Within the frail cohort, the incidence of dysphagia was greatest among patients who underwent ACDF (ACDF: 9.9% vs. PCDF: 3.1%, *p* < 0.001), Table 3. There were no significant differences in incidence of any complication (ACDF: 18.8% vs. PCDF: 20.6%, *p* = 0.289) or the number of complications (*p* = 0.182) between procedure types, Table 3. Within the severely frail cohort, the incidence of any complication (ACDF: 25.3% vs. PCDF: 31.2%, *p* = 0.011) and the number of complications (*p* = 0.020) was greater among patients undergoing PCDF, Table 3. The incidence of dysphagia was greatest in patients undergoing ACDF (ACDF: 13.1% vs. PCDF: 3.9%, *p* < 0.001), Table 3.

### 3.4. Postoperative Inpatient Outcomes

Comparing the frailty cohorts, healthcare resource utilization varied significantly between cohorts. The mean LOS (*p* < 0.001) and NRD rate (*p* < 0.001) increased significantly with increasing frailty, Table 4. Within the pre-frail cohort, mean LOS (ACDF: 2.3 ± 2.7 days vs. PCDF: 4.1 ± 4.1 days, *p* < 0.001) and NRD rate (ACDF: 9.0% vs. PCDF: 28.7%, *p* < 0.001) were significantly greater among patients who underwent PCDF compared to patients who underwent ACDF, Table 4. Within the frail cohort, mean LOS (ACDF: 2.7 ± 3.3 days vs. PCDF: 5.0 ± 7.8 days, *p* < 0.001) and NRD rate (ACDF: 14.7% vs. PCDF: 39.6%, *p* < 0.001) were significantly greater among patients who underwent PCDF compared to patients who underwent ACDF, Table 4. Within the severely frail cohort, mean LOS (ACDF: 4.0 ± 5.6 days vs. PCDF: 6.2 ± 6.1 days, *p* < 0.001) and NRD rate (ACDF: 22.6% vs. PCDF: 46.4%, *p* < 0.001) were significantly greater among patients who underwent PCDF compared to patients who underwent ACDF, Table 4.

### 3.5. Multivariate Regression for Healthcare Utilization for ACDF

On multivariate analysis for extended LOS in patients who underwent ACDF, extended LOS increased with frailty status compared to pre-frail, frailty [OR (CI): 1.39 (1.15, 1.68), *p* < 0.001], and severe frailty [OR (CI): 2.25 (1.83, 2.76), *p* < 0.001], Table 5. On multivariate analysis for NRD in patients who underwent ACDF, NRD increased with frailty status compared to pre-frail, frailty [OR (CI): 1.49 (1.21, 1.84), *p* < 0.001], and severe frailty [OR (CI): 2.22 (1.77, 2.79), *p* < 0.001], Table 5.

### 3.6. Multivariate Regression for Healthcare Utilization for PCDF

On multivariate analysis for extended LOS in patients who underwent PCDF, extended LOS increased with frailty status compared to pre-frail, frailty [OR (CI): 1.58 (1.23, 2.03), *p* < 0.001], and severe frailty [OR (CI): 2.45 (1.88, 3.20), *p* < 0.001], Table 6. On multivariate analysis for NRD in patients who underwent PCDF, NRD increased with frailty status compared to pre-frail, frailty [OR (CI): 1.55 (1.26, 1.90), *p* < 0.001], and severe frailty [OR (CI): 1.63 (1.28, 2.07), *p* < 0.001], Table 6.

## 4. Discussion

In this retrospective national database study of 37,990 patients who underwent ACDF or PCDF for CSM, we found that patient frailty independently impacts ACDF and PCDF patients similarly, with an overall increase in healthcare resource utilization within the PCDF patients.

The decision to pursue anterior vs. posterior approaches for CSM is multifactorial and has been discussed previously, as delineated in Table 7. In a retrospective study of 140 patients who underwent anterior or posterior decompression for CSM, Audat et al. found that neck disability index (NDI) score and radiographic outcomes were similar between approach cohorts at five year follow-up [27]. Similarly, in a systematic review of eight level III retrospective cohort studies of patients who underwent decompression for CSM, Lawrence et al. demonstrated that while incidence of infection and dysphagia varied between approaches, there was no clear generalizable advantage to either an anterior approach (discectomy or corpectomy) or a posterior approach (laminectomy only, laminectomy with fusion, or laminoplasty) for multilevel CSM with respect to treatment effectiveness or safety and suggested that an individualized decision-making strategy is necessary to select the preferred treatment for each patient [28]. Furthermore, in a meta-analysis of ten non-randomized controlled trials evaluating the clinical efficacy of anterior and posterior approaches for multilevel CSM, Luo et al. determined that no clear conclusion could be reached regarding which approach is more efficacious for multilevel CSM [29]. A review comparing the anterior and posterior approaches for degenerative cervical myelopathy by Kato et al. suggests selecting an approach based on radiographic features contributing to spinal cord compression [30]. For example, in cases of CSM due to disc herniation, an anterior approach may be preferred, though a posterior approach may be ideal in CSM related to ligamentum flavum ossification due to the relative ease of accessing these structures with the respective approaches [30,31,32].

Other studies have suggested that the anterior approach is the preferred approach in most cases of CSM [33,34,35,36]. In a retrospective cohort study of 89 patients who underwent anterior or posterior decompression surgery for CSM at a single institution, Hitchon et al. recommended the anterior approach due to the benefits in hospital LOS and restoration of physiological cervical lordosis compared to the posterior approach, despite similar outcomes in complications, quality of life, and sagittal balance [33]. Similarly, in a multi-institutional database study of 1151 patients who underwent decompression surgery for CSM, Wilkerson et al. observed that after controlling for baseline differences between patients who underwent anterior or posterior surgery, the anterior approach was associated with greater improvements in NDI score at both the 3 month and 12 month follow-ups [34]. A similar conclusion was made in a meta-analysis of 25 studies including 1843 patients who underwent decompression for CSM [35]. However, no large, prospective randomized clinical trials (RCT) have been published in the literature. In a small, single institutional RCT of 68 patients who underwent surgery for multilevel degenerative cervical myelopathy, El-Ghandour et al. found that the anterior approach was superior with respect to postoperative pain, NDI score, and hospital LOS, though the posterior approach was associated with reduced incidence of postoperative dysphagia and shorter operative time [36]. In a more recently published RCT of 163 patients randomized to anterior surgery (ACDF) or posterior surgery (laminectomy with fusion or laminoplasty) at fifteen large hospitals in the U.S. and Canada, Ghogawala et al. found that while postoperative complications were significantly more common in the anterior surgery group (including dysphagia, new neurological deficit, 30-day readmission, and reoperation), there were no significant differences in patient-reported outcomes at one year follow-up [37]. Our study demonstrated similar findings with regards to rates of dysphagia for the ACDF cohort; however, the length of hospitalizations and total number of complications were increased in the PCDF cohort in comparison. Additional studies may be warranted to better elucidate the clinical and radiographical criteria for the varying approaches.

Frailty has been suggested to contribute to the progression of CSM and suboptimal improvement in patient outcomes following both anterior and posterior decompressive approaches [38]. In a retrospective NSQIP database study of 17,662 patients who underwent ACDF for a number of indications, Momtaz et al. found that patient frailty was associated with postoperative AEs, readmission, and reoperation following ACDF [39]. Additionally, in a multi-institutional NSQIP database study of 41,369 patients who underwent surgery for CSM from 2010 to 2018, Wilson et al. found that mFI-defined frailty was a more effective predictor of poor outcomes than age alone, with increasing frailty being associated with increased incidence of perioperative complications, increased hospital LOS, and NRD [23]. Similarly, in a retrospective NIS database cohort study of 29,305 patients who underwent ACDF for CSM, Elsamadicy et al. found that patient frailty, was associated with greater AE risk, prolonged hospital LOS and increased rate of NRD [40]. Similar findings have been shown with posterior approach as well. In a retrospective cohort study of 6965 patients who underwent ACDF or posterior cervical fusion, Shin et al. demonstrated that mFI-11-defined frailty was an independent predictor of Clavien-Dindo grade IV complications (life-threatening single/multiorgan dysfunction requiring intermediate care or intensive care unit management) in both the ACDF and posterior cervical fusion cohorts [41]. Similarly, in a retrospective cohort study of 165 patients who underwent PCDF at an academic medical center from 2014 to 2020, Lambrechts et al. found that while mFI-11-defined frailty did not significantly impact complication rates, 90-day readmission rates, reoperation rates, or patient-reported outcome measures, patients with severe frailty were significantly more likely to experience longer LOS and NRD [42]. Additionally, in a retrospective NSQIP study of 5627 patients who underwent posterior cervical fusion, Medvedev et al. found that frailty was predictive of blood transfusion, prolonged extubation greater than 48 h, reintubation, readmission, and reoperation [43]. In the present study utilizing the mFI-11 to identify frailty, we found that increasing frailty influenced increasing postoperative AEs and experiencing greater hospital LOS and rates of NRD. Given the increased risk of suboptimal outcomes in frail patients who undergo decompression for CSM, preoperative identification of frailty and patient preoptimization are necessary.

As increased frailty has a negative impact on clinical outcomes, these outcomes may then disproportionately affect patients who are socioeconomically disadvantaged. Our study found that the frail and severely frail cohorts contained a significantly higher proportion of non-white patients, patients in the lowest income quartile, and those covered by government insurance. Moreover, on multivariate analysis, non-white race was independently associated with greater odds of increased LOS and NRD, while being in the highest income quartile or having private insurance was associated with lower odds of increased LOS and NRD among patients undergoing ACDF. Among patients undergoing PCDF, black race and having Medicaid insurance were associated with higher odds of increased LOS. Thus, our present study indicates that not only do socioeconomically disadvantaged groups have greater frailty scores but also that they are associated with poorer hospital outcomes. Because of the impact on clinical outcomes, it is crucial that healthcare delivery continues to be equitable and target patients’ individual needs.

With the increased identification of risk factors associated with poor outcomes following spine surgery, many have sought to develop methods to improve perioperative patient optimization to reduce complications and improve efficiency of healthcare delivery while maintaining patient safety [44]. These methods, commonly referred to as enhance recovery after surgery (ERAS) protocols, include optimizing nutrition, incorporating multimodal pain control, encouraging early ambulation, and limiting urinary catheterization, with the goal of improving patient outcomes [45]. In spine surgery, implementation of ERAS protocols has been shown to reduce opioid consumption, hospital LOS, and accelerate time to ambulation following surgery [44,45,46,47]. More specifically, some have sought to determine how implementation of ERAS protocols may affect outcomes following cervical spine surgery [48,49]. In a retrospective study of 33 patients who underwent ACDF or cervical arthroplasty for numerous indications, Soffin et al. found that implementation of a multidisciplinary ERAS protocol was feasible and safe, with no 90-day readmissions [48]. In a retrospective study of 404 propensity score-matched patients who underwent ACDF for degenerative cervical radiculopathy at a single institution, Debono et al. observed that ERAS protocol implementation was associated with a significant reduction in hospital LOS, without increasing risk of postoperative complications [49]. To our knowledge, no studies have assessed the effectiveness of an ERAS protocol for North American patients with high frailty scores who undergo cervical spine surgery specifically for CSM. Given that in our study, we found that severely frail patients had longer hospital stays and greater rates of non-routine discharge, utilizing protocols that would encourage early ambulation, effective pain management, and shorter length of stay may prove especially useful for this population. Thus, additional studies are necessary to determine whether an ERAS protocol similar to those utilized in other populations would be effective in the CSM population and how patient frailty may affect ERAS protocol effectiveness.

This study has some limitations that may have implications on interpretation and generalizability. Although all variables were recorded preoperatively, intraoperatively, and postoperatively, they were reviewed retrospectively and, thus, are subject to the limitations of retrospective analyses. Given that the diagnoses in the NIS database are organized by diagnostic codes, some collected data have been misclassified, incomplete, or incorrectly identified in the database. Additionally, while the NIS database offers a relatively high sample size, some granular patient- and hospital-level details may be missed. Moreover, we are not able to control for the degree of neurological injury and radiographic stenosis, two key aspects of the clinical presentation that may impact surgical decision-making, postoperative recovery, and length of stay, and non-routine discharge. Similarly, the data regarding unplanned hospital readmission and reoperation rates are not available in the NIS. Furthermore, the impact of frailty is assessed after the decision for surgical intervention is made, and we are limited by the NIS database to assess the varying degrees of frailty in all patients considered for surgery. Because we only included patients who underwent surgical procedures, we cannot generalize to all patients with CSM, many of whom may not have received surgery and only received medical management due to their high frailty status. Finally, we are also limited by a potential selection bias that may have occurred, and our sample may not represent the entire population as a whole. Despite these limitations, this study sheds light on the impact that preoperative frailty has on complications and healthcare resource utilization in patients who undergo anterior or posterior decompression and fusion for CSM.

## 5. Conclusions

Our study suggests that preoperative frailty may impact outcomes after surgical treatment for CSM, with more frail patients having greater health care utilization and a higher rate of adverse events. Patients undergoing PCDF increased health care utilization, compared to ACDF; thus, severely frail patients undergoing PCDF tended to have the longest length of stay and highest rate of non-routine discharge. Additional prospective studies are necessary to identify the optimum surgical approach in frail patients incorporating clinical and radiographic metrics.

## Figures and Tables

**Table 1:** Patient Demographics and Hospital Characteristics Among Pre-Frail, Frail, and Severely Frail Patients Undergoing ACDF and PCDF.

	Pre-Frail (n = 16,665)			Frail (n = 12,985)			Severely Frail (n = 8340)			*p*-Value (Totals)
	ACDF (n = 11,655)	PCDF (n = 5010)	*p*-Value	ACDF (n = 8470)	PCDF (n = 4515)	*p*-Value	ACDF (n = 5170)	PCDF (n = 3170)	*p*-Value	
**Age (Years)**										
Mean ± SD	61.06 ± 11.11	64.67 ± 10.96	**<0.001**	63.07 ± 10.00	66.21 ± 10.25	**<0.001**	64.44 ± 9.46	67.74 ± 9.29	**<0.001**	**<0.001**
**Female (%)**	50.4	43.2	**<0.001**	45.3	38.0	**<0.001**	37.8	33.1	**0.057**	**<0.001**
**Race (%)**			**<0.001**			**0.007**			0.182	**0.042**
White	76.0	69.1		73.4	67.2	**<0.001**	73.6	69.6		
Black	13.8	17.7		15.9	21.0		16.8	19.4		
Hispanic	5.8	6.5		6.3	7.4		5.2	7.1		
Other	4.3	6.6		4.3	4.4		4.5	3.9		
**Income Quartile (%)**			**0.002**			**0.007**			0.547	**<0.001**
0–25th	26.7	23.1		31.8	29.4		33.2	30.7		
26–50th	27.3	24.2		26.5	22.0		27.4	27.5		
51–75th	25.7	27.1		24.8	27.0		24.8	24.8		
76–100th	20.3	25.6		16.9	20.7		14.6	17.0		
**Healthcare Coverage (%)**			**<0.001**			**<0.001**			**0.019**	**<0.001**
Medicare	44.8	55.7		51.4	60.9		60.8	67.5		
Medicaid	9.8	8.3		10.8	9.4		11.4	11.2		
Private Insurance	38.9	30.2		30.6	23.5		21.6	15.6		
Other	6.5	5.8		7.2	6.2		6.2	5.7		
**Hospital Bed Size (%)**			**0.014**			**<0.001**			0.079	**<0.001**
Small	21.3	18.1		21.1	17.1		16.0	14.7		
Medium	26.2	22.8		29.1	22.1		27.0	22.6		
Large	52.5	59.2		49.8	60.8		57.1	62.8		
**Hospital Region (%)**			**<0.001**			**<0.001**			**<0.001**	**<0.001**
Northeast	11.7	19.1		11.8	17.1		9.3	17.4		
Midwest	17.3	22.5		17.8	25.2		23.4	28.9		
South	48.5	35.9		52.1	38.1		50.5	36.8		
West	22.5	22.6		18.3	19.6		16.8	17.0		
**Hospital Type (%)**			**<0.001**			**<0.001**			**<0.001**	**<0.001**
Rural	2.3	2.1		2.8	2.1		3.3	5.5		
Urban Non-Teaching	23.6	11.9		20.8	9.9		22.4	11.4		
Urban Teaching	74.1	86.0		76.3	88.0		74.3	83.1		

Bold signifies statistical significance of *p*-value < 0.05.

**Table 2:** Admission and Patient Comorbidities Among Pre-Frail, Frail, and Severely Frail Patients Undergoing ACDF and PCDF.

	Pre-Frail (n = 16,665)			Frail (n = 12,985)			Severely Frail (n = 8340)			*p*-Value (Totals)
	ACDF (n = 11,655)	PCDF (n = 5010)	*p*-Value	ACDF (n = 8470)	PCDF (n = 4515)	*p*-Value	ACDF (n = 5170)	PCDF (n = 3170)	*p*-Value	
Functional status	2.2	4.2	**0.001**	5.5	10.9	**<0.001**	16.0	22.1	**0.002**	**<0.001**
Hypertension	60.5	59.6	0.610	80.5	79.4	0.519	86.9	83.3	**0.039**	**<0.001**
History of COPD or pneumonia	4.1	3.4	0.368	14.7	12.3	0.094	41.9	39.1	0.264	**<0.001**
Impaired sensorium	18.1	14.3	**0.007**	30.4	29.0	0.456	50.7	49.5	0.647	**<0.001**
Diabetes	11.2	13.9	**0.032**	46.9	44.6	0.255	63.2	58.4	**0.046**	**<0.001**
History of MI	2.4	2.8	0.438	15.3	17.2	0.224	46.4	47.9	0.540	**<0.001**
History of CHF	0.4	0.7	0.315	3.1	2.3	0.245	14.9	17.5	0.173	**<0.001**
History of stroke	0.2	0.5	0.168	1.2	1.8	0.274	5.1	6.3	0.313	**<0.001**
History of TIA	0.1	0.0	0.257	0.1	0.0	0.466	0.1	0.5	0.128	0.135
History of PCI	0.0	0.1	0.127	0.1	0.0	0.465	0.3	0.5	0.544	**0.001**
PVD	0.7	0.6	0.674	2.2	2.5	0.614	6.8	8.0	0.327	**<0.001**
Deficiency anemias	0.9	1.5	0.098	1.3	2.1	0.115	1.8	3.8	**0.015**	**<0.001**
Alcohol use	1.0	1.4	0.362	2.4	4.3	**0.008**	3.8	6.3	**0.014**	**<0.001**
Paralysis	1.8	6.8	**<0.001**	3.2	6.6	**<0.001**	4.1	8.0	**0.001**	**0.001**
Affective disorder	26.9	25.5	0.407	29.5	25.0	**0.015**	34.6	33.1	0.534	**<0.001**
Smoking history	12.3	9.3	**0.015**	19.5	18.1	0.379	30.7	31.4	0.758	**<0.001**
Cervicalgia	0.9	1.8	**0.042**	0.7	0.7	0.898	0.6	0.3	0.446	**0.027**
Headache	3.9	3.5	0.527	3.4	2.7	0.291	3.4	1.4	**0.013**	0.077
Dorsalgia	11.6	11.4	0.871	12.0	12.3	0.852	12.2	11.4	0.619	0.766

Bold signifies statistical significance of *p*-value < 0.05.

**Table 3:** Adverse Events Among Pre-Frail, Frail, and Severely Frail Patients Undergoing ACDF and PCDF.

	Pre-Frail (n = 16,665)			Frail (n = 12,985)			Severely Frail (n = 8340)			*p*-Value (Totals)
	ACDF (n = 11,655)	PCDF (n = 5010)	*p*-Value	ACDF (n = 8470)	PCDF (n = 4515)	*p*-Value	ACDF (n = 5170)	PCDF (n = 3170)	*p*-Value	
Acute kidney injury	1.4	3.6	**<0.001**	1.7	3.4	**0.005**	4.7	6.8	0.088	**<0.001**
Acute post-hemorrhagic anemia	2.4	5.1	**<0.001**	3.1	7.4	**<0.001**	3.1	7.4	**<0.001**	**0.005**
Post-operative pain	1.7	3.5	**0.002**	1.4	2.8	**0.009**	2.2	5.4	**0.001**	**0.005**
Acute respiratory failure	1.4	1.8	0.403	2.3	2.4	0.834	6.2	6.2	0.975	**<0.001**
Circulatory complications	0.3	0.9	**0.023**	0.4	0.3	0.751	1.3	1.7	0.424	**<0.001**
Mechanical ventilation	0.7	0.9	0.518	1.5	1.1	0.375	3.3	2.8	0.612	**<0.001**
Nervous system complications	1.2	2.6	**0.003**	0.9	2.1	**0.009**	0.9	2.1	**0.040**	0.597
UTI	1.5	2.8	**0.017**	2.1	5.0	**<0.001**	3.4	6.0	**0.010**	**<0.001**
Sepsis	0.1	0.7	**0.002**	0.4	0.3	0.928	1.3	1.1	0.780	**<0.001**
Dysphagia	8.3	2.3	**<0.001**	9.9	3.1	**<0.001**	13.1	3.9	**<0.001**	**<0.001**
Any complication	15.4	19.3	**0.008**	18.8	20.6	0.289	25.3	31.2	**0.011**	**<0.001**
Number of complications			**0.021**			0.182			**0.020**	**<0.001**
0	84.4	80.7		81.2	78.8		75.2	69.1		
1	12.7	14.7		14.9	15.4		16.8	21.8		
2	2.0	3.6		3.0	4.1		4.5	6.3		
≥3	0.9	1.0		1.0	1.7		3.5	2.8		

Bold signifies statistical significance of *p*-value < 0.05.

**Table 4:** Postoperative Inpatient Outcomes Among Pre-Frail, Frail, and Severely Frail Patients Undergoing ACDF and PCDF.

	Pre-Frail (n = 16,665)			Frail (n = 12,985)			Severely Frail (n = 8340)			*p*-Value (Totals)
	ACDF (n = 11,655)	PCDF (n = 5010)	*p*-Value	ACDF (n = 8470)	PCDF (n = 4515)	*p*-Value	ACDF (n = 5170)	PCDF (n = 3170)	*p*-Value	
**Length of stay (days)**										
Mean ± SD	2.3 ± 2.7	4.1 ± 4.1	**<0.001**	2.7 ± 3.3	5.0 ± 7.8	**<0.001**	4.0 ± 5.6	6.2 ± 6.1	**<0.001**	**<0.001**
Median [IQR]	1 [1, 2]	3 [2, 5]	**<0.001**	2 [1, 3]	3 [2, 6]	**<0.001**	2 [1, 5]	4 [3, 8]	**<0.001**	**<0.001**
**Disposition (%)**			**<0.001**			**<0.001**			**<0.001**	**<0.001**
Routine	90.8	71.1		85.2	59.9		76.2	53.0		
Non-Routine	9.0	28.7		14.7	39.6		22.6	46.4		
Other	0.2	0.2		0.2	0.4		1.2	0.6		

Bold signifies statistical significance of *p*-value < 0.05.

**Table 5:** Multivariate Regression for Healthcare Utilization for ACDF.

	Extended LOS (>3 Days)	*p*-Value	Non-Routine Discharge	*p*-Value
mFI-11				
Pre-Frail	Reference			
Frail	**1.39 (1.15, 1.68)**	**<0.001**	**1.49 (1.21, 1.84)**	**<0.001**
Severely Frail	**2.25 (1.83, 2.76)**	**<0.001**	**2.22 (1.77, 2.79)**	**<0.001**
Age	1.00 (0.99, 1.01)	0.382	**1.04 (1.03, 1.06)**	**<0.001**
Female sex	0.99 (0.84, 1.16)	0.888	0.96 (0.81, 1.15)	0.691
Race				
White	Reference			
Black	**2.08 (1.67, 2.58)**	**<0.001**	**2.29 (1.80, 2.91)**	**<0.001**
Hispanic	**1.72 (1.27, 2.34)**	**<0.001**	**1.62 (1.13, 2.31)**	**0.008**
Other	**1.75 (1.19, 2.56)**	**0.004**	**1.48 (1.00, 2.19)**	**0.049**
Income Quartile				
0–25th	Reference			
26–50th	0.93 (0.76, 1.15)	0.510	1.05 (0.82, 1.33)	0.673
51–75th	0.84 (0.68, 1.04)	0.117	1.02 (0.79, 1.32)	0.867
76–100th	**0.68 (0.52, 0.89)**	**0.005**	**0.73 (0.54, 0.99)**	**0.040**
Healthcare Coverage				
Medicare	Reference			
Medicaid	1.20 (0.89, 1.61)	0.225	1.05 (0.71, 1.53)	0.821
Private Insurance	**0.71 (0.58, 0.88)**	**0.002**	**0.58 (0.45, 0.76)**	**<0.001**
Other	1.05 (0.75, 1.46)	0.792	**0.57 (0.37, 0.86)**	**0.007**
Hospital Bed Size				
Small	Reference			
Medium	**1.39 (1.05, 1.83)**	**0.020**	Removed	*-*
Large	**2.00 (1.56, 2.57)**	**<0.001**	Removed	*-*
Hospital Region				
Northeast				
Midwest	Removed	*-*	Removed	*-*
South	Removed	*-*	Removed	*-*
West	Removed	*-*	Removed	*-*
Hospital Type				
Rural	Reference			
Urban Non-Teaching	Removed	*-*	0.91 (0.51, 1.63)	0.747
Urban Teaching	Removed	*-*	1.08 (0.62, 1.89)	0.783
Fusion Levels				
One level	Reference			
Two or more	Removed	*-*	Removed	*-*
Number of Complications				
0	Reference			
1	**4.79 (3.98, 5.78)**	**<0.001**	**2.82 (2.27, 3.51)**	**<0.001**
2	**15.16 (10.19, 22.57)**	**<0.001**	**5.49 (3.75, 8.02)**	**<0.001**
>2	**50.14 (23.33, 107.78)**	**<0.001**	**13.74 (8.02, 23.54)**	**<0.001**
Length of Stay	-	-	-	-

Removed refers to variables that were included in the univariate regression analysis but did not meet entry criteria (*p* < 0.1) for the multivariate. Bold signifies statistical significance of *p*-value < 0.05.

**Table 6:** Multivariate Regression for Healthcare Utilization for PCDF.

	Extended LOS (>6 Days)	*p*-Value	Non-Routine Discharge	*p*-Value
mFI-11				
Pre-Frail	Reference			
Frail	**1.58 (1.23, 2.03)**	**<0.001**	**1.55 (1.26, 1.90)**	**<0.001**
Severely Frail	**2.45 (1.88, 3.20)**	**<0.001**	**1.63 (1.28, 2.07)**	**<0.001**
Age	**1.02 (1.01, 1.04)**	**0.002**	**1.06 (1.05, 1.07)**	**<0.001**
Female sex	0.96 (0.77, 1.20)	0.719	**1.26 (1.04, 1.52)**	**0.016**
Race				
White	Reference			
Black	**1.89 (1.45, 2.45)**	**<0.001**	**1.71 (1.35, 2.17)**	**<0.001**
Hispanic	1.35 (0.92, 1.98)	0.122	1.37 (0.96, 1.96)	0.079
Other	1.19 (0.72, 1.96)	0.498	1.06 (0.71, 1.59)	0.766
Income Quartile				
0–25th	Reference			
26–50th	Removed	-	Removed	*-*
51–75th	Removed	-	Removed	*-*
76–100th	Removed	-	Removed	*-*
Healthcare Coverage				
Medicare	Reference			
Medicaid	**1.75 (1.15, 2.68)**	**0.010**	0.95 (0.65, 1.38)	0.786
Private Insurance	1.14 (0.82, 1.57)	0.441	**0.59 (0.45, 0.77)**	**<0.001**
Other	1.47 (0.91, 2.35)	0.113	**0.60 (0.39, 0.92)**	**0.020**
Hospital Bed Size				
Small	Reference			
Medium	**1.47 (1.01, 2.16)**	**0.047**	**1.44 (1.04, 1.99)**	**0.026**
Large	**1.58 (1.13, 2.23)**	**0.008**	**1.40 (1.05, 1.87)**	**0.021**
Hospital Region				
Northeast	Reference			
Midwest	Removed	*-*	0.76 (0.57, 1.02)	0.071
South	Removed	*-*	**0.67 (0.52, 0.87)**	**0.003**
West	Removed	*-*	**0.65 (0.49, 0.87)**	**0.004**
Hospital Type				
Rural	Reference			
Urban Non-Teaching	Removed	-	Removed	-
Urban Teaching	Removed	-	Removed	-
Fusion Levels				
One level	Reference			
Two or more	Removed	-	Removed	-
Number of Complications				
0	Reference			
1	**3.51 (2.75, 4.51)**	**<0.001**	**2.63 (2.08, 3.33)**	**<0.001**
2	**7.13 (4.69, 10.82)**	**<0.001**	**4.09 (2.66, 6.30)**	**<0.001**
>2	**39.60 (14.85, 105.56)**	**<0.001**	**8.29 (3.96, 17.35)**	**<0.001**
Length of Stay	Removed	-	Removed	-

Bold signifies statistical significance of *p*-value < 0.05.

**Table 7:** Review of Anterior and Posterior Surgical Approaches to Treatment of CSM.

Authors	Study Type	Key Findings
Wilson JRF et al., 2020 [23]	Retrospective study	MFI-defined frailty was a more effective predictor of poor outcomes than age alone, with increasing frailty being associated with increased incidence of perioperative complications, increased hospital LOS, and NRD.
Audat ZA et al., 2018 [27]	Retrospective study	NDI score and radiographic outcomes were similar between anterior and posterior approach cohorts at five year follow-up.
Lawrence BD et al., 2013 [28]	Systematic review	There was no clear generalizable advantage to either an anterior or posterior approach for multilevel CSM with respect to treatment effectiveness or safety.
Luo J et al., 2015 [29]	Meta-analysis	No clear conclusion could be reached regarding which approach is more efficacious for multilevel CSM.
Kato S et al., 2018 [30]	Review	Authors suggest choosing a surgical approach based on radiographic features contributing to spinal cord compression.
Zhu B et al., 2013 [31]	Systematic review and meta-analysis	In cases of CSM due to disc herniation, an anterior approach may be preferred.
Feng F et al., 2016 [32]	Systematic review and meta-analysis	A posterior approach may be ideal in CSM related to ligamentum flavum ossification due to the relative ease of accessing these structures, although the anterior approach had better overall postoperative neural function.
Hitchon PW et al., 2019 [33]	Retrospective cohort study	The anterior approach saw benefits in hospital LOS and restoration of physiologic cervical lordosis compared to the posterior approach, despite similar outcomes in complications, quality of life, and sagittal balance.
Wilkerson CF et al., 2022 [34]	Retrospective study	The anterior approach was associated with greater improvements in NDI score at both the 3-month and 12-month follow-ups.
Chen Z et al., 2017 [35]	Meta-analysis	The anterior approach was associated with better postoperative neurologic function.
El-Ghandour NMF et al., 2020 [36]	RCT	The anterior approach was superior with respect to postoperative pain, NDI score, and hospital LOS, though the posterior approach was associated with reduced incidence of postoperative dysphagia and shorter operative time.
Ghogawala Z et al., 2021 [37]	RCT	While postoperative complications were significantly more common in the anterior surgery group (including dysphagia, new neurological deficit, 30-day readmission, and reoperation), there were no significant differences in patient-reported outcomes at one year follow-up.
Badhiwala JH et al., 2020 [38]	Post hoc analysis	Frailty and comorbidities negatively impact functional outcomes in CSM patients undergoing decompression.
Momtaz D et al., 2022 [39]	Retrospective study	Patient frailty was associated with postoperative AEs, readmission, and reoperation following ACDF.
Elsamadicy AA et al., 2023 [40]	Retrospective cohort study	Patient frailty was associated with greater AE risk, prolonged hospital LOS, increased rate of NRD, and higher admission costs.
Shin JI et al., 2017 [41]	Retrospective cohort study	MFI-11-defined frailty was an independent predictor of life-threatening single/multiorgan dysfunction in both the ACDF and posterior cervical fusion cohorts.
Lambrechts MJ et al., 2017 [42]	Retrospective cohort study	While mFI-11-defined frailty did not significantly impact complication rates, 90-day readmission rates, reoperation rates, or patient-reported outcome measures, patients with severe frailty were significantly more likely to experience longer LOS and NRD.
Medvedev G et al., 2016 [43]	Retrospective study	In patients who underwent posterior cervical fusion, frailty was predictive of blood transfusion, prolonged extubation greater than 48 h, reintubation, readmission, and reoperation.
Young R et al., 2020 [44]	Observational	Patients undergoing elective cervical or lumbar surgeries had lower postoperative opioid use and LOS after ERAS implementation.
Bansal T et al., 2022 [45]	Narrative review	Across many spine surgeries, ERAS protocols reduce health care utilization and involve multimodal pain management and early mobilization.
Elsarrag M et al., 2019 [46]	Systematic review	ERAS protocols may decrease LOS, costs, and pain in spine surgery.
Debono B et al., 2019 [47]	Retrospective study	Use of ERAS protocols in patients with ACDF, anterior lumbar interbody fusion, and posterior lumbar fusion led to decreased LOS and improved patient satisfaction.
Soffin EM et al., 2019 [48]	Retrospective study	Implementation of a multidisciplinary ERAS protocol was feasible and safe, with no 90-day readmissions, among patients who underwent ACDF or cervical arthroplasty.
Debono B et al., 2021 [49]	Retrospective study	ERAS protocol implementation was associated with a significant reduction in hospital LOS, without increasing risk of postoperative complications.

## Data Availability

Data used for this study is from the National Inpatient Sample, publicly available from the Healthcare Cost and Utilization Project. More information can be found at http://www.hcup-us.ahrq.gov/nisoverview.jsp (accessed on 20 November 2023).

## Appendix A

**Table A1:** Inclusion and Exclusion Criteria.

Diagnosis or Procedure	ICD-10 Codes
**Inclusion**	
ACDF	0RG10A0, 0RG20A0
Cervical Laminectomy	00NW0ZZ
Cervical Internal Fixation	0RH104Z
**Exclusion**	
Traumatic spinal fracture	S12.0x, S12.1x, S12.2x, S12.4x, S12.5x, S12.6x, S12.8x, S17.x, S22.0x, S32.0x, S32.1x
Neoplasms of vertebral column, spinal cord, meninges of spinal cord	C41.2, C41.9, C70.1, C70.9, C72.0, C72.1, C72.9
Percutaneous/Endoscopic Posterior cervical fusion, Posterior approach to Anterior Column	0RG207J, 0RG20AJ, 0RG20JJ, 0RG20KJ, 0RG2371, 0RG237J, 0RG23AJ, 0RG23J1, 0RG23JJ, 0RG23K1, 0RG23KJ, 0RG2471, 0RG247J, 0RG24AJ, 0RG24J1, 0RG24JJ, 0RG24K1, 0RG24KJ, 0RG107J, 0RG10AJ, 0RG10JJ, 0RG10KJ, 0RG1371, 0RG137J, 0RG13AJ, 0RG13J1, 0RG13JJ, 0RG13K1, 0RG13KJ, 0RG1471, 0RG147J, 0RG14AJ, 0RG14J1, 0RG14JJ, 0RG14K1, 0RG14KJ

## Appendix B

**Table A2:** Modified Frailty Index.

Comorbidities in mFI-11	ICD-10 Codes
Functional status	H54, R26.0-R26.9, R27.0-R27.9, R41, R41.81, R54, S72, Z73, Z74.1, Z73.6, Z74
History of hypertension requiring medication	I10, I11, I12, I13, I15
History of chronic obstructive pulmonary disease or pneumonia	J12, J13, J14, J15, J16, J17, J18, J43, J44
History of impaired sensorium	A81.0, F00-F03, F01, F04, F05, F06, F10, F11-F19, G20, G30, H35
History of diabetes mellitus	E10, E11, E13, E14
History of myocardial infarction	I21, I22, I25
History of congestive heart failure	I50, U80.2
History of stroke with neurologic deficit	I61, I63, I69
History of TIA or stroke without neurological deficit	G45
History of PCI, angina, or stenting	I20
History of peripheral vascular disease or ischemic rest pain	I70.2, I73, I77.9, I77.1

## Appendix C

**Table A3:** ICD-10 Codes for Patient Characteristics, Intraoperative Variables, and Adverse Events.

Diagnosis or Procedure	ICD-10 Codes
Affective disorder	F30.x, F31.x, F32.x, F33.x, F34.x, F41.x
Smoking history	F17210, F17213, F17290, F17293
Cervicalgia	M542
Headache	G441, R51, G43909, G43919, G43901, G43911
Dorsalgia	M54, M540, M5400, M5401, M5402, M5403, M5404, M5405, M5406, M5407, M5408, M5409M541, M5410, M5411, M5412, M5413, M5414, M5415, M5416, M5417, M5418, M542, M543, M5430, M5431, M5432, M544, M5440, M5441, M5442, M545, M546, M548, M5481, M5489, M549
Fusion of 1 cervical level	0RG10A0, 0RG1071, 0RG10J1, 0RG10K1
Fusion of 2 or more cervical levels	0RG20A0, 0RG2071, 0RG20J1, 0RG20K1
CSF leak or dural tear	G96.0, G96.1, G96.11
Acute kidney injury	N170, N171, N172, N178, N179
Acute post-hemorrhagic anemia	D62
Post-operative pain	G8918
Acute respiratory failure	J810, J952, J9582, J95821, J95822, J95831, J960, J9600, J9601, J9602, J962, J9620, J9621, J9622
Circulatory complications	I97, I970, I971, I9711, I97110, I97111, I9712, I97120, I97121, I9713, I97130, I97131, I9719, I97190, I97191, I972, I973, I974, I9741, I97410, I97411, I97418, I9742, I975, I9751, I9752, I976, I9761, I97610, I97611, I97618, I9762, I97620, I97621, I97622, I9763, I97630, I97631, I97638, I9764, I97640, I97641, I97648, I977, I9771, I97710, I97711, I9779, I97790, I97791, I978, I9781, I97810, I97811, I9782, I97820, I97821, I9788, I9789
Mechanical ventilation	09HN7BZ, 09HN8BZ, 0BH13EZ, 0BH17EZ, 0BH18EZ, 5A19054, 5A1935Z, 5A1945Z, 5A1955Z
Nervous system complications	G97.82
UTI	N39.0
Sepsis	A41, A410, A4101, A4102, A411, A412, A413, A414, A415, A4150, A4151, A4152, A4153, A4159, A418, A4181, A4189, A419
Dysphagia	R13, R130, R131, R1310, R1311, R1312, R1313, R1314, R1319
Mechanical device complication	T84216, T84216A, T84218, T84218A, T84226, T84226A, T84228, T84228A, T84296, T84296A, T84298, T84298A, T8431, T84310, T84310A, T84318, T84318A, T8432, T84320, T84320A, T84328, T84328A, T8439, T84390, T84390A, T84398, T84398A
Displacement of internal fixation device of vertebrae	T84226A
